# Energy Gaps in BN/GNRs Planar Heterostructure

**DOI:** 10.3390/ma14175079

**Published:** 2021-09-05

**Authors:** Jinyue Guan, Lei Xu

**Affiliations:** 1 Xinjiang Key Laboratory of Solid State Physics and Devices, Xinjiang University, Urumqi 830046, China; chinaguanjinyue@163.com; 2 Center for Theoretical Physics, School of Physical Science and Technology, Xinjiang University, Urumqi 830046, China

**Keywords:** BN/GNRs heterostructure, local potential, lattice match, band gap

## Abstract

Using the tight-binding approach, we study the band gaps of boron nitride (BN)/ graphene nanoribbon (GNR) planar heterostructures, with GNRs embedded in a BN sheet. The width of BN has little effect on the band gap of a heterostructure. The band gap oscillates and decreases from 2.44 eV to 0.26 eV, as the width of armchair GNRs, $n_{A}$, increases from 1 to 20, while the band gap gradually decreases from 3.13 eV to 0.09 eV, as the width of zigzag GNRs, $n_{Z}$, increases from 1 to 80. For the planar heterojunctions with either armchair-shaped or zigzag-shaped edges, the band gaps can be manipulated by local potentials, leading to a phase transition from semiconductor to metal. In addition, the influence of lattice mismatch on the band gap is also investigated.

## 1. Introduction

Since its discovery in 2004, graphene has become a research field of tremendous interest [1,2], and has also led to the rise in two-dimensional materials. Its unique composition and electronic energy bands give graphene a series of attractive physical properties, such as high carrier mobility, atomic thinness and linear dispersion [3,4]. Although graphene has many excellent properties, the characteristic of zero band gap limits its application in the semiconductor industry. Graphene can be chemically modified by heteroatom insertion to modulate its photoelectric properties or chemical activity [5]. Nitrogen doping is one of the most popular chemical modifications of graphene, as it has great potential for applications, ranging from supercapacitors, to fuel cells, to hydrogen storage materials [6,7]. Cutting graphene in specific patterns can form graphene nanoribbons (GNRs), quasi-one-dimensional graphene nanostructures, which can exhibit either quasi-metallic or semiconducting behavior, depending on their specific chirality, including width, lattice orientation and edge geometry [8]. There are two different groups of GNRs, categorized according to the edge termination types, i.e., zigzag and armchair GNRs [9]. Both of them have been shown to have band gaps [10]. 

With the continuous development of the two-dimensional materials field, many graphene-like materials have been discovered in succession, for example, hexagonal boron nitride (h-BN), tungsten disulfide, silylene and germanene. One of the most notable two-dimensional materials is h-BN, which has a crystal structure very similar to graphene. This is because it is a wide-band-gap semiconductor, regardless of the shape of the edge [11,12].

The lattice constant of h-BN differs by only 1.8% compared to that of graphene, and small lattice differences tend to form a heterojunction between graphene and h-BN. One method is to stack them together to form a van der Waals heterojunction [13,14,15]. This structure has a smaller systemic dimension, and it produces interesting physical properties that are not available in a single material [16]. Another method is to combine graphene with h-BN to form a planar heterojunction [17,18,19,20]. The band gap and magnetism of this structure can be precisely designed with proper control [20,21,22].

Recently, Wang et al. successfully prepared chiral, controllable, monolayer GNRs via a two-step growth method in experiments. They directly embedded monolayer GNRs in BN nano-trenches by modifying the ratio of working-gases [23], and achieved edge control of graphene domains on h-BN [24,25]. This structure shows excellent electronic properties and has attracted considerable attention.

In this paper, we report results from a study that explored the band structure of lattice-matched GNRs embedded in BN-sheet-forming planar heterostructures via the tight-binding approach. It was found that band gaps change with the width of GNRs. For both armchair GNRs (AGNRs) and zigzag GNRs (ZGNRs) embedded in a BN sheet, the band structures can be controlled by local potentials, and a similar phase transition can be achieved.

## 2. Models and Methods

The tight-binding Hamiltonians for monolayer graphene and BN can be written as follows:

(1) $$\begin{array}{c} H_{G}=-t_{1}\sum_{\langle i,j\rangle }\left(C_{i}^{†}C_{j}+H.c.\right)\;, \end{array}$$

(2) $$\begin{array}{c} H_{BN}=-t_{2}\sum_{\langle i,j\rangle }\left(C_{i}^{†}C_{j}+H.c.\right)+\sum_{i}E_{i}C_{i}^{†}C_{i}, \end{array}$$

where $C_{i}^{†}\left(C_{i}\right)$ is an electron creation (annihilation) operator on site *i*, $t_{1}$ and $t_{2}$ are the nearest-neighbor hoppings of monolayer graphene and BN, respectively. $E_{i}$ is the on-site energy, which is $E_{B}$ and $E_{N}$ for boron and nitrogen atoms, respectively. $H_{G}$ and $H_{BN}$ are the Hamiltonians of monolayer graphene and BN that only consider the nearest-neighbor hopping.

The Hamiltonian of BN/GNRs is as follows:

(3) $$\begin{array}{c} H=H_{G}+H_{BN}+\sum_{\langle i,j\rangle }\left(t_{BN-G}C_{i}^{†}C_{j}+H.c.\right), \end{array}$$

where the third term describes the interlayer interaction between the BN layer and the graphene layer. $t_{BN-G}$ is the nearest-neighbor hopping of the boron nitride and graphene heterojunction, containing two kinds of hopping integral, $t_{C-N}$ and $t_{C-B}$. $t_{C-N}$ and $t_{C-B}$ are the nearest-neighbor hoppings of C-N and C-B atoms at the interface of the heterojunction. The tight binding parameters are listed in Table 1, and these parameters refer to the DTF data [22,26,27]. 

**Figure 1:**
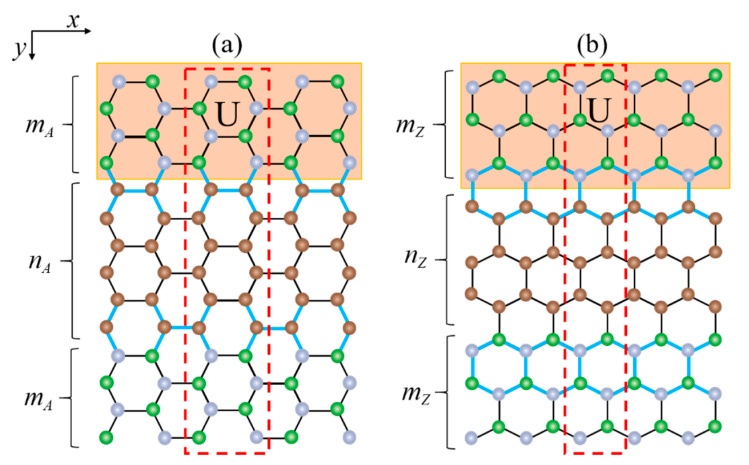
Schematic of (**a**) BN/AGNRs and (**b**) BN/ZGNRs heterostructure. Carbon, nitrogen and boron atoms are denoted by brown, green and gray solid circles, respectively. $n_{A}$ and $m_{A}$ represent the number of armchair chains in graphene and BN sheets, while $n_{Z}$ and $m_{Z}$ represent zigzag ones. A unit cell is indicated by the red dashed box. The local potential, U, is only applied in the upper BN region indicated in brown. The lattice-distorted bonds at the interface are shown in blue.

As shown in Figure 1, we named AGNRs embedded in a BN sheet as ($m_{A}$,$n_{A}$) BN/AGNRs. $m_{A}$ is the number of armchair chains in the BN sheet on both sides, and $n_{A}$ is the number of armchair chains in GNRs. Similarly, ZGNRs embedded in BN are named ($m_{Z}$,$n_{Z}$) BN/ZGNRs, where $m_{Z}$ is the number of zigzag chains in the BN sheet on both sides, and $n_{Z}$ is the number of zigzag chains in the GNRs. The total width of this heterojunction structure can be changed by adjusting the three regions, respectively.

In this paper, we discuss two different structures of BN/AGNRs and BN/ZGNRs, but will only consider a case where the width of the BN sheet is the same on both sides for simplicity. Firstly, the width, $n_{A}\left(n_{Z}\right),$ of graphene in the middle is unchanged, while the width, $m_{A}\left(m_{Z}\right),$ of BN on both sides is changed. Secondly, the width $m_{A}\left(m_{Z}\right)$ of BN on both sides is fixed and the width $n_{A}\left(n_{Z}\right)$ of graphene is changed to study the band structures. The lattice constant mismatch between graphene and BN is only 1.8%, we therefore, first consider that BN and graphene have the same lattice constant, *τ* = 2.46 Å. The effects of lattice mismatches are discussed later.

## 3. Results and Discussion

### 3.1. The Band Structure of GNRs and BNNRs

**Figure 2:**
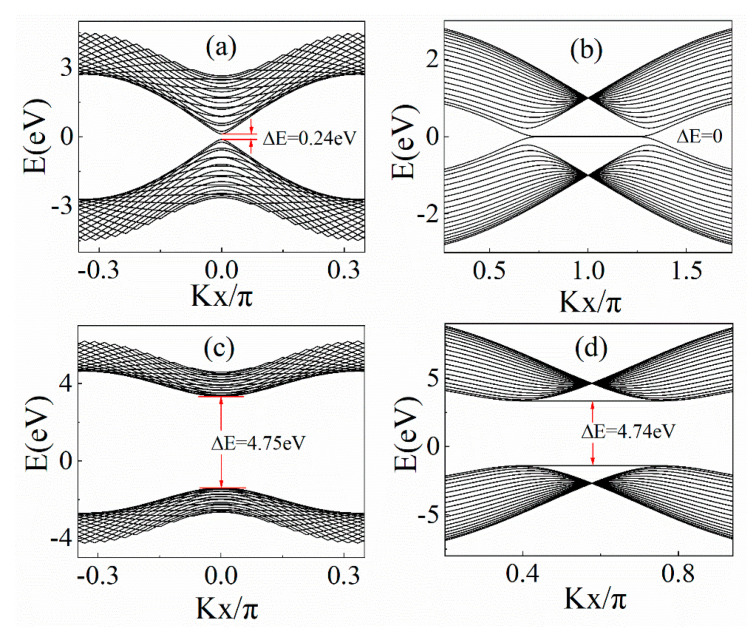
Band structures of (**a**) AGNR, (**b**) ZGNR, (**c**) armchair BNNR and (**d**) zigzag BNNR with 20 chains.

We calculated the band structures of GNRs and boron nitride nanoribbons at two different edge types. As shown in Figure 2a,c, the GNRs and boron nitride nanoribbons (BNNRs) with the armchair-shaped edge have band gaps, and the band gaps’ Δ*E* is 0.24 eV and 4.75 eV, respectively. The band gap Δ*E* was calculated by subtracting the maximum valence from the minimum conduction band. For the zigzag-edged GNRs and BNNRs, the band structures are shown in Figure 2b,d, and the band gaps were 0 eV and 4.74 eV, respectively.

### 3.2. The Band Structure of BN/GNRs

**Figure 3:**
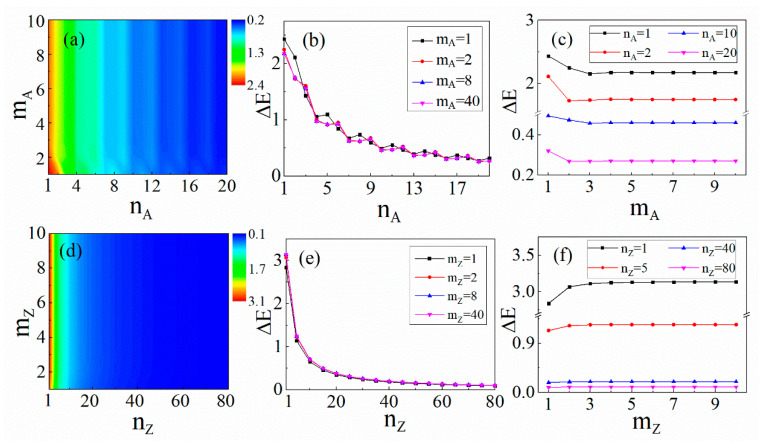
Contour plots for the band gaps of (**a**) BN/AGNRs and (**d**) BN/ZGNRs in the $n_{A}$-$m_{A}$ plane. The color scale indicates the magnitude of the gap in units of electron volt. The band gaps of (**b**) BN/AGNRs and (**e**) BN/ZGNRs as a function of $n_{A}$ and $n_{Z}$. The band gaps of (**c**) BN/AGNRs and (**f**) BN/ZGNRs as a function of $m_{A}$ and $m_{Z}$.

For BN/AGNRs, we took $n_{A}$ from 1 to 20 and $m_{A}$ from 1 to 10, and calculated the corresponding band gaps, as shown in Figure 3a. As can be seen from the phase diagram, the band gap is never zero, which indicates that BN/AGNR is a semiconductor.

It was also found that the width of GNRs obviously affects the band gap, but the width of BN barely does so, as illustrated in Figure 3b. For certain numbers of armchair chains, $m_{A}$ = 1, 2, 8 and 40, it can be seen that the band gap oscillated and decreased from 2.44 eV to 0.26 eV, as the width of AGNRs increased from 1 to 20. Once $m_{A}\geq 2$, the band gap remained the same for different values of $m_{A}$, which can clearly be seen in Figure 3c. This means that the width of BN has little effect on the band gap of a heterostructure. Note that the band gap with $m_{A}=1$ was obviously different from the others, probably because the full hexagonal lattice was not formed in this case and the quantum size effect was dominant. 

For BN/ZGNRs, we also calculated the band gaps, as shown in Figure 3d. Taking the number of BN chains, $m_{Z}$ = 1, 2, 8 and 40, the band gaps showed almost the same variation pattern and gradually decreased from 3.13 eV to 0.09 eV, as the width of GNRs increased from 1 to 80, as depicted in Figure 3e. In contrast, when the width of GNRs remained unchanged, the band gap showed a completely different pattern as the width of the BN increased. When the width of GNRs was small ($n_{Z}$ = 1 and 5), the band gap increased slightly with the increase in the BN width and then remained unchanged. For wider GNRs ($n_{Z}$ = 40 and 80), the band gap hardly changed with the increase in the BN width. In such cases, the size of the band gap was dominated by the width of the GNRs: the wider the GNRs, the smaller the band gap. However, due to the interaction between graphene and BN, the band gap was close to zero, but not zero.

Clearly, the band structure of BN/GNRs is different from that of both graphene and BN, and the BN/GNRs planner heterojunction is a semiconductor, whose band gap is between that of graphene and BN.

In the case of both BN/AGNRs and BN/ZGNRs, when the width of BN is small, the band gap changes obviously, which may be caused by the quantum size effect. Once the width of BN is large, the band gap will not change with the width of BN, but will be determined by GNRs. Thus, it is appropriate to take the number of the BN chain as $m_{A}$ = 8–10 hereafter.

**Figure 4:**
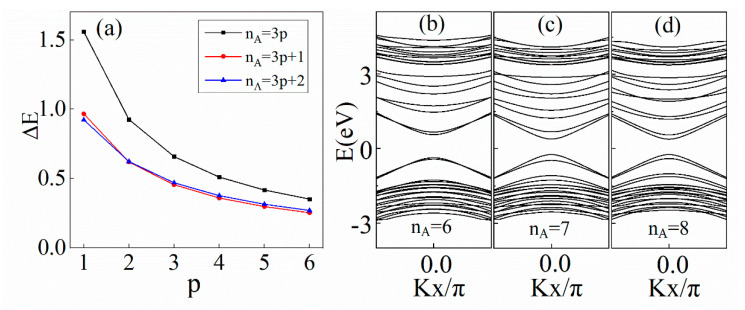
(**a**) Band gaps of (8,$n_{A}$) BN/AGNRs as a function of *p*. Calculated band structures of (8,$n_{A}$ ) BN/AGNRs for (**b**) $n_{A}$ = 6 (**c**) $n_{A}$ = 7 and (**d**) $n_{A}$ = 8.

As shown in Figure 4, the band gaps of (8, $n_{A}$) BN/AGNRs have an oscillatory behavior with period p = 3. Therefore, the band gap behavior can be divided into three cases, $n_{A}$ = 3p, 3p + 1 and 3p + 2, where *p* is a positive integer. At smaller sizes (p ≤ 2), there is a hierarchy of gap size: $\Delta E_{3p}>\Delta E_{3p+1}>\Delta E_{3p+2}$. For example, the band gaps of (8, 6), (8, 7) and (8, 8) BN/AGNRs are 0.928 eV, 0.625 eV and 0.622 eV, respectively. At larger sizes (p > 2), the gap size hierarchy is changed to $\Delta E_{3p}>\Delta E_{3p+2}>\Delta E_{3p+1}$. This result is similar to that of graphene nanoroads in BN sheets [28], but it may seem different from the band gap hierarchy in [29]: $\Delta E_{3q}>\Delta E_{3q+1}>\Delta E_{3q+2}$, where $N_{A}$ = 3q, 3q + 1 and 3q + 2, and q is a positive integer. This is because different parameters have been used. Here, $n_{A}$ represents the number of armchair chains in graphene, whereas $N_{A}$ represents the number of carbon columns in graphene [28], that is, $n_{A}$ = $2N_{A}$. By comparing the available data, it was found that the band gaps obey the same pattern of change. According to our calculations, (8, $n_{A}$) BN/AGNRs ($n_{A}$ = 1–20) are all semiconductors with direct band gaps. This semiconducting behavior can be thought of as quantum confinement.

### 3.3. The Effect of Lattice Mismatch

Now, we discuss the effects of lattice distortion. The lattice constants of BN and graphene are 2.5 Å and 2.46 Å, respectively, corresponding to the bond lengths $a_{B-N}$ = 1.45 Å and $a_{C-C}$ = 1.42 Å. GNRs embedded in BN-sheet-forming planar heterostructures can lead to lattice mismatches at the interface, due to the difference between the lattice constants of these two materials. In the above calculation, we show an ideal model where the lattice mismatches are neglected. Moreover, this also does not consider the difference in the lattice parameter between the periodic geometry and the nanoribbon geometry; that is, the influence of the edge on the lattice parameter is not considered.

**Figure 5:**
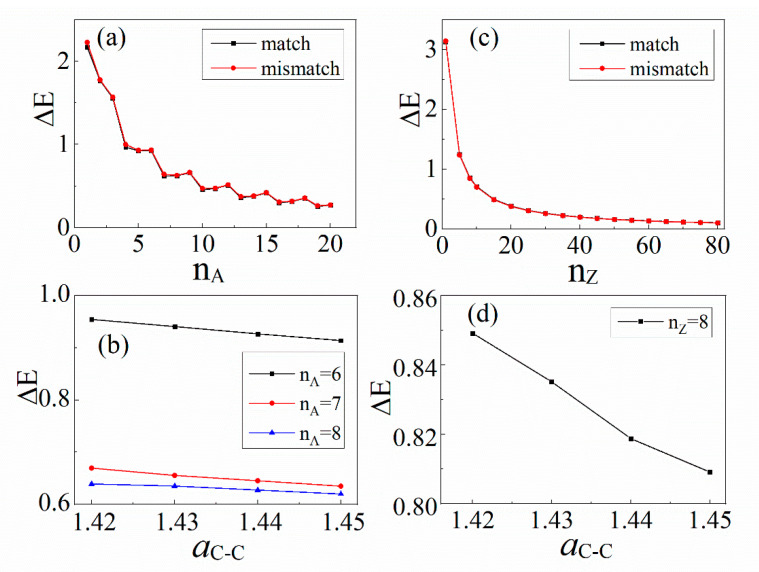
Band gap variations of (**a**) BN/AGNRs and (**c**) BN/ZGNRs for lattice-matched and lattice-mismatched models. Band gap variations of (**b**) BN/AGNRs and (**d**) BN/ZGNRs, as a function of lattice parameters.

In general, hydrogen passivation is used to reduce the lattice distortion at the edge [10,17,21,28,29,30,31]. Therefore, we only discuss the effects of lattice mismatches. There is a tensile strain at the interface of a BN/graphene/BN structure, and the bond lengths of C-C atoms at the interface are slightly prolonged [24] and less than 1.8%, while the bond lengths of C-N and C-B atoms do not change. Based on this, we take the prolonged bond length of C-C atoms at the interface, $a_{C-C}$ = 1.43 Å, and the corresponding hopping integral, $t_{1}$ = 2.76 eV, as an example. The band gaps of the planner heterojunctions with and without lattice mismatch are presented in Figure 5a,c. It is easy to see that the pattern of change of the band gaps is almost identical.

When the lattice constant changes at the interface, the corresponding hopping integral also changes proportionately. In Figure 5b,d, we observe that the band gaps for both BN/AGNRs and BN/ZGNRs decrease almost linearly as the bond lengths of C-C atoms at the interface increase. Even when the bond lengths of C-C atoms increase to 1.45 Å, the maximum decrease in the band gap is only 6.6%. That is to say, the lattice distortion only slightly reduces the band gap, but does not change the overall band structure. The band gap is always more than zero, and the heterojunctions belong to the semiconductors. Therefore, we assert that the lattice mismatches have little effect on the band gap, and it is appropriate for us to choose the lattice-matched mode.

### 3.4. The Influence of Local Potential

To better control the band gap, we explored the band structures of BN/AGNRs in the presence of local potentials. Since, based on the calculation results, the band gap of the heterojunction is insensitive to the local potential in the graphene region, we only added the local potential in the BN region. When the local potential was applied to the B and N atoms in the upper BN region (the brown region in Figure 1), the band gap could be adjusted by altering the local potential.

**Figure 6:**
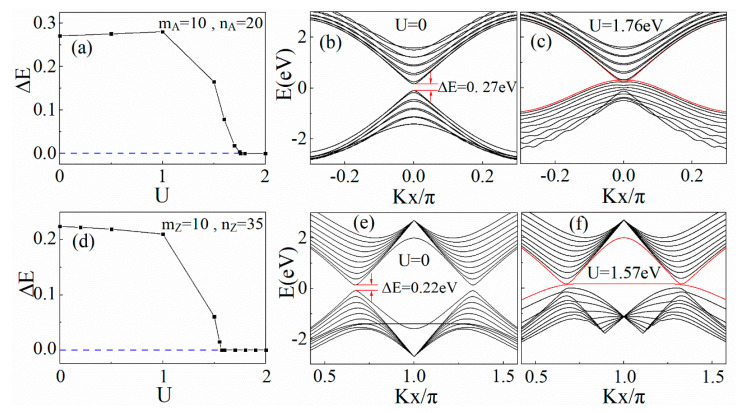
Energy gaps as a function of local potential for the (**a**) BN/AGNRs and (**d**) BN/ZGNRs. The horizontal blue dashed line represents the zero band gap. Band structures of (10,20) BN/AGNRs with local potentials (**b**) *U* = 0 and (**c**) *U* = 1.76 eV. Band structures of (10,35) BN/ZGNRs with local potentials (**e**) *U* = 0 and (**f**) *U* = 1.57 eV.

Figure 6a,d show how the band gap changed along with the local potential. As the local potential increased, the band gap of BN/AGNRs with $m_{A}$ = 10 and $n_{A}$ = 20 increased slightly at first, then decreased sharply, and, finally, decreased to zero. The band structure without local potential is depicted in Figure 6b, where an obvious band gap, Δ*E* = 0.27 eV, is found. The band gap vanished at a critical local potential, *U* = 1.76 eV, and the heterostructure transitioned into metal. When *U* was further enhanced, the band gap remained at zero, as shown in Figure 6c.

For BN/ZGNRs with $m_{Z}$ = 10 and $n_{Z}$ = 35, as the local potential increased, the band gap decreased slowly at first, then decreased very quickly, and, finally, decreased to zero. The band structures of (10,35) BN/ZGNRs are shown in Figure 6e. There was a band gap, Δ*E* = 0.22 eV at *U* = 0, which is consistent with the previous DFT and tight-binding results [32]. When the local potential increased to a critical value, *U* = 1.57 eV, the valance and conduction bands met, and the gap vanished, as shown in Figure 6f. This indicates that the system underwent a phase transition from the semiconductor phase to the metallic phase.

## 4. Conclusions

In summary, we studied the band structure of lattice-matched GNRs embedded in h-BN. The findings clearly indicate that the width of BN has little effect on the band gap. The band gap of BN/AGNRs oscillates and decreases with the increase in the width of AGNRs, while that of BN/ZGNRs gradually decreases with the increase in the width of ZGNRs. The effects of lattice mismatches, which have little effect on the band gap, have also been considered. When the BN region suffers the applied local potential, the heterojunction of BN/GNRs can change from the state with a band gap to the state without a band gap. This means that the band gap can be controlled by local potentials, giving rise to a phase transition from semiconductor to metal. We hope our results will be helpful for future applications relating to electronic devices. They may also provide basic building blocks for integrated circuits.

## Figures and Tables

**Table 1:** Value of the tight-binding parameters. The energy is in the unit of electron volts (eV).

$t_{1}$	$t_{2}$	$t_{C-N}$	$t_{C-B}$	$E_{B}$	$E_{N}$
2.7	2.8	2.6	2.89	3.34	−1.4

## Data Availability

The data presented in this study are contained within the article and are available on request from the corresponding author.
